# Role of Square Flap in Post Burn Axillary Contractures

**Published:** 2017-09

**Authors:** Durga Karki, Ravi Prakash Narayan

**Affiliations:** Department of Burns, Plastic and Maxillofacial Surgery, Vardhaman Mahavoir Medical Collefe and Safdarjung Hospital, New Delhi, India

**Keywords:** Burn, Axillary, Contracture, Square flap

## Abstract

**BACKGROUND:**

Post-burn contractures are a commonly encountered problem and many techniques have been described in their treatment. Z-plasties are the commonest local flap procedure done for linear bands with adjacent healthy tissue. Our aim was to assess the use of square flap technique in axillary contractures.

**METHODS:**

Ten patients with type I and II axillary contractures underwent release by the square flap technique. All cases were followed up for at least one year and analysed for range of motion and aesthetic outcome.

**RESULTS:**

All cases achieved full range of movement postoperatively with no recurrence during follow up period and a good cosmetic outcome.

**CONCLUSION:**

Square flap was shown to be a reliable technique for mild to moderate axillary contractures of the anterior or posterior axillary folds even when there is significant adjacent scarring of chest wall or back of types I and II.

## Introduction

Burns are still a devastating condition in emergency medicine in both developed and developing countries, while the depth and size of the burn are important factors in morbidity and mortality of burn injuries.^1^ For those who survive, scarring is the most persisting problem, so there is always a hope to decrease the problems related to formation of scars. Also, inhalation injuries and other complications influence the outcome.^2^ The incidence of burns in India is very high and due to various socioeconomic factors, many patients go on to develop complications like burn contractures. Burn contracture of the axilla is an especially debilitating problem as shoulder movement impact the ability to locate the hand in space to perform activities of daily living.^3^

At the time of injury, the victim usually observes a protective posture in which the arms are tightly adducted and flexed. As a result, the anterior and posterior axillary folds are usually affected often sparing the axillary fossa.^4^ The best treatment is prevention i.e., good primary care, splinting and regular exercises. But once a contracture has developed, early surgical release should be done.^4^^-^^6^ Axillary contractures are classified on the basis of site of contracture, involvement of axillary fossa and adjacent scarring, and the surgical treatment varies with each type.^5^^,^^7^^-^^9^

Hanumadass *et al.*^4^ has classified axillary contracture as Type I: Linear web at anterior or posterior fold with minimal adjacent scarring and no involvement of the hair bearing area; Type II: Scar contracture involving either anterior or posterior axillary folds with adjacent skin scarring, but sparing the hair bearing area.; Type III: Linear webs at both anterior and posterior axillary folds with or without adjacent scarring, but sparing the hair bearing area; and Type IV: Contracture of one or both folds, diffuse adjacent scarring and involving the hair bearing area.^4^

Release and skin grafting is simple to design and perform and can be done in all types of axillary contractures, but there is risk of failure of graft take, re-contracture, long term splint, absence of suitable donor sites in severe burn cases and painful donor sites.^5^^,^^9^ Use of flaps over joints decrease the recurrence rate, because of inclusion of normal skin and subcutaneous tissue, and splinting need not be done for more than two weeks.^10^^-^^12^ Local flaps are best suited for contractures having some adjacent healthy tissue that can be used. These include skin elongation procedures such as Z plasty and its modifications, transposition flaps and propeller flaps.^5^

Z plasty is the most commont performed local skin flap technique^6^ in type I contractures, wherein there is a linear web with healthy adjacent skin.^2^^,^^7^^,^^8^ Transposition flaps require healthy adjacent chest wall, back or arm skin that can be transposed after release. Propeller flaps use adipocutaneous tissue from the axillary hair bearing region based on a random subcutaneous pedicle rotated 90 degrees to resurface the contracture at the axilla.^13^ The disadvantage is that skin grafts are usually required after flap rotation.^13^

Square flap is a local tissue transposition technique first described by Limberg in 1963, later modified by Hyakusoku and Fumiri in 1987 as a method to increase the distance between two points.^14^ It is an useful method for scar contracture, various clefts and cryptotia. It gives a theoretical lengthening of 2.80 times the original length and does not the cut across the axillary hair bearing region like Zplasty.^14^^,^^15^ In thias study, we have assessed the outcome of the square flap technique in type I and II axillary contractures of various severity.

## MATERIALS AND METHODS

All post-burn axillary contractures that presented to our outpatient department from July 2011 to March 2015 were considered for the study. Contractures of anterior or posterior axillary fold with and without adjacent scarring but sparing of axillary hair bearing region i.e., type I and II contractures of Hanumadass classification^4^ were selected. Preoperatively, patients’ demographic data, history regarding the cause of burns, and the course of treatment were recorded. Type of contracture and its severity noted in terms of maximum range of shoulder abduction were noted. 

**Fig. 1:**
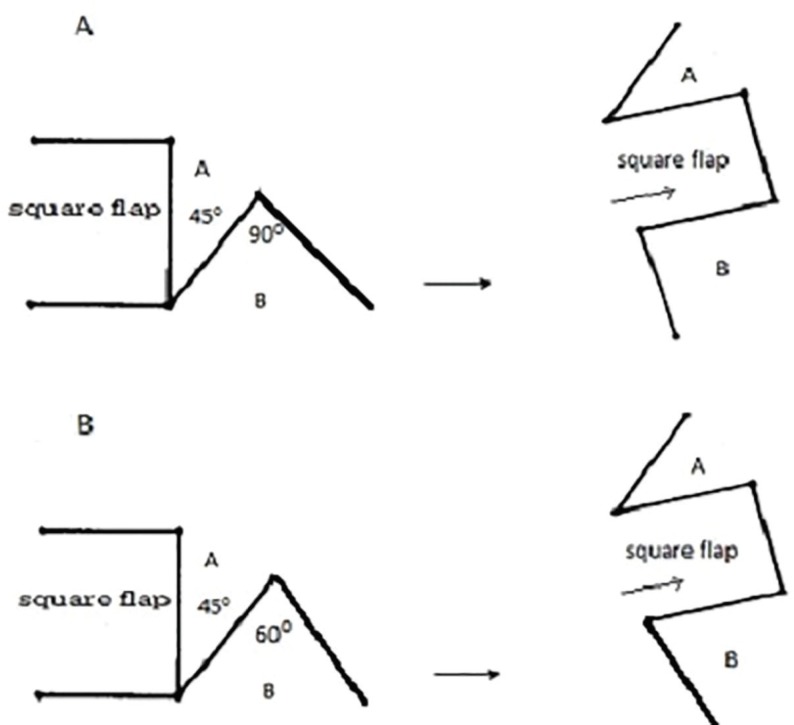
**A:** Schematic diagram of square flap described by Hyakusoku with the first triangular flap having an angle (angle A) of 45^o^ and the second triangular flap having an angle (angle B) of 90^o^. **B:** Schematic diagram of a modified square flap with the first triangular flap having an angle (angle A) of 45^o^ and the second triangular flap having an angle (angle B) of 60^o^

All surgeries were performed under general anesthesia. A square was designed adjacent to the contracture on the side of the axillary fossa and two adjacent triangular flaps on the other side of the contracture. The lengths of the sides of the square and triangular flaps were kept equal. The angle of the first triangular flap A was kept at 45 degrees and the second flap B ranging from 60 to 90 degrees (Figure 1). Lesser angles were used in patients with hyperthrophic scarring of the adjacent anterior chest wall or back. After the incisions were made, the contracted scar tissue was released. The square flap was then advanced across the area of contracture and the adjacent triangular flaps were rotated and positioned one on each side of the advanced square flap proximally and distally. 

Postoperative splint was given for 7 days. Sutures were removed after complete healing at 10-15 days. Any postoperative complications were noted. Regular physiotherapy was done postoperatively. Follow up visits were scheduled for minimum period of 1 year and range of motion and photographs were taken at each visit. A few of the illustrated cases were mentioned.

**Fig. 2:**
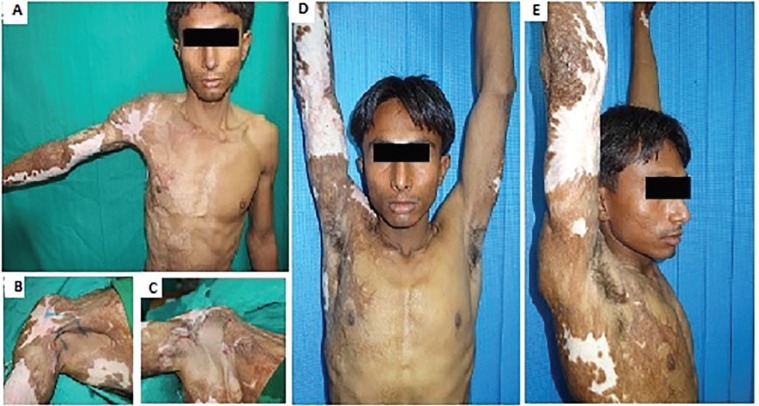
**A:** Preoperative view of right axillary contracture type 1a; **B:** Preoperative marking of square flap; **C:** Sutured flaps after advancement of square flap and transposition of triangular flaps; **D, E:** Postoperative front and lateral view at 1.5 years follow up respectively showing full abduction of right shoulder

Case 1 was a 20-year old male presented after 2 years of thermal burns with contracture of anterior axillary fold of right axilla and abduction restricted beyond 80^o ^(Figure 2). Square flap was advanced from axillary fossa, flap A transposed distal to square flap and flap B transposed proximal to square flap. 

**Fig. 3:**
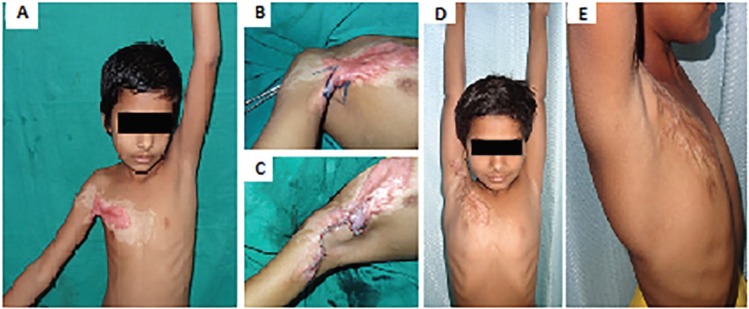
**A:** Preoperative view of right axillary contracture type 1a; **B:** Preoperative marking of square flap; **C: **Sutured flaps after advancement of square flap and transposition of triangular flaps; **D, E:** Postoperative front and lateral view at 1 years follow up respectively showing full abduction of right shoulder

A postoperative abduction of 180^o^ was achieved and maintained at follow up after 1.5 years. Case 2 was a 8-year old child presented 9 months after thermal burns with contracture of anterior part of right axilla with hypertrophic scarring of adjacent chest wall. Maximum shoulder abduction was 35^0^. Contracture was released, square flap advanced from axillary fossa and triangular flap A transposed proximal to square flap and triangular flap B transposed distal to square flap (Figure 3). A postoperative abduction of 180^o^ was achieved, and the outcome was cosmetically satisfactory. Patient was followed up for about 1 year and the abduction was maintained at 180^o^.

## RESULTS 

The square flap was used in a total of ten patients in the age group ranging from 6 to 35 years, of which seven were males and three were females. The mean age of the patients was 19.2 years with male:female ratio of 7:3. The mean duration of contracture was 1.8 years. The cause of contracture was thermal burns in all patients. In all cases, the wounds healed with conservative management and patients did not follow any splinting and exercise protocols. Six were involving the anterior axillary fold and four involving the posterior axillary fold. Eight of the cases had associated adjacent scarring (type II) and two had linear webs with healthy surrounding skin. 

No skin grafts were needed for cover of the raw area created after release. In one case, there was tip necrosis of triangular flap; but it healed well with conservative management. No other complications were noted post-operatively in any of the patients. All patients were followed up for minimum one year. Pre-operatively, axillary abduction ranged from 35 to 90 degrees with a mean of 62.5 degrees. Complete range of motion (180^o^) was achieved in all cases and no recurrences were observed (Table 1). Since the square flap was advanced from the axillary fossa, the hair bearing skin was not divided across and transposed, giving a good cosmetic appearance post-operatively. There was also good colour and texture match.

**Table 1:** Data of patients included in the study

**No.**	**Age**	**Gender**	**Type of axillary contracture**	**Complication**	**Follow up (years)**	**Preoperative range of motion**	**Range of motion 1 year post-op**
1	4	Female	Type1a (Rt)	Nil	2	80^ o^	180^o^
2	25	Male	Type 1b(Lt)	Nil	1.8	90^ o^	180^o^
3	20	Male	Type1a (Rt)	Nil	1.5	80^ o^	180^o^
4	18	Male	Type1a (Rt)	Tip necrosis	1.6	60^ o^	180^o^
5	30	Male	Type1b (Rt)	Nil	1	90^ o^	180^o^
6	28	Female	Type1a (Lt)	Nil	1.8	90^ o^	180^o^
7	35	Male	Type1a (Rt)	Nil	1.7	60^ o^	180^o^
8	24	Male	Type1b (Lt)	Nil	1.3	85^ o^	180^o^
9	8	Female	Type1a (Rt)	Nil	1	35^ o^	180^o^
10	22	Male	Type1b (Rt)	Nil	1	55^ o^	180^o^

## DISCUSSION

With improved surgical techniques, refinements in the algorithms for management of axillary contractures are in order.^5^^,^^7^^,^^8^ Level 1 and 2 evidences (randomized studies) are lacking in the field of treatment of burn contractures.^6^ Each case should be individualised based on patient factors (nature of contracture, patient compliance, socioeconomic factors), surgeon factors (skills and experience) in order to achieve desired results in the least resource intense manner.^16^^,^^17^ Hanumadass *et al.* recommended Z plasties for type I contractures and release and skin grafting for types II, III and IV (based on his classification).^4^

Sakr *et al.* recommended multiple Z plasties for linear contractures; local transposition and advancement flaps for moderate localized contracture bands with healthy adjacent skin; scapular and parascapular flaps, when local flaps are unfeasible due to scarring; and skin grafts when scapular and parascapular skin is scarred.^8^ According to Ogawa *et al.*’s study, skin grafting should be done if the contracture is small, flat and not involving the fat layer of the axilla; local flaps when contracture involves anterior or posterior axillary folds and spares axillary fossa with minimal adjacent scarring; propeller flap when both folds are involved but fossa is uninvolved; axial local flap (latissimus dorsi flap, parascapular flap, superficial cervical artery flap and bilateral scapular combined flap) when there is adjacent scarring of chest, back or upper arm; free flap and scarring flap when contracture is extensive.^5^

When using local flaps for contractures of both anterior and posterior folds, flaps should be staged.^6^ A single large Z plasty provides good lengthening but large flaps are prone to more transverse tension. Modifications such as multiple Z plasty in series, four-flap, five- flap, and six- flap Z plasties gave lengthening with lesser transverse tension. However, when the angles of the flap are narrow especially in scarred skin, they are prone to tip necrosis. Another disadvantage of Z plasty in axillary contracture is that it divides the hair bearing area of the axilla and displaces a part of it anteriorly over the chest wall, which looks bad cosmetically.^3^^,^^9^

Theoretically, Z plasty and its various modifications can achieve a lengthening of 75 to 150% (1.75 to 2.5 times gain in length of central limb), but the actual lengthening achieved may be lesser in vivo.^18^ There are no established guidelines as to which Z plasty should be used where, nor is it specified how severe a contracture can be corrected using Z plasty. Unless the scar is a discrete band, they will not provide the desired result without skin grafting.^19^ Square flap is considered a three flap modification of Z plasty that combines flap transposition and advancement. In the original square flap design described before, the design contained a square and two triangular flaps, both having an acute angle.^14^

This design was modified by making one of the triangular flaps a right angled one, and was demonstrated better lengthening as compared to Limberg’s design (Figure 1). Also, an added advantage was that the suture line was not parallel to the line of lengthening. The lengthening resulting from Z plasty, its various modifications and square flap were assessed geometrically before realising that square flap gave the maximum lengthening. When the angle of the second triangle flap was 90 degrees, it gave a lengthening of 2.8 times, in comparison to Z plasty which gave a maximum lengthening of 2.24 times. Square flap provided a gain in length of almost 180% as per simple geometric analysis and about 90% elongation when skin elasticity and 3D deformation were taken into consideration by computer aided analysis. The gain in length was more compared to any other methods derived from Z plasty.^14^^,^^15^

More recent studies using stereometric geometric modelling revealed that square flap yields a larger flap area, higher length breadth ratio compared to Z plasties and is associated with the lowest physiological tension, which means that the deformity of the adjacent skin and the dependence on the laxity of the adjacent skin is minimal.^20^ In addition to contractures, square flap can be used in various clefts (cleft earlobe, cleft palate), cryptotia, and epicanthal folds.^14^^,^^21^^,^^22^ Several modifications of the square flap have been proposed to better suit its use in clefts, epicanthal folds and contractures.^14^^,^^22^^,^^23^

We used the square flap technique is cases with significant adjacent scarring. In a few of our cases, the second triangular flap was taken at 60 degrees instead of 90 degrees, as the severe hypertrophic scarring precluded transposition of a larger angled flap (Figure 1). We achieved good lengthening without tension in all cases, even when initial range of motion was as low as 35^o^ and abduction of 180 degrees was maintained at one year follow up in all cases. Recurrence is unlikely to occur later, if the range of motion is preserved till one-year post-surgery.^3^

The design is simple and easy to replicate. The contracture is cut across at a point and the square advancement flap breaks the line of the contracture. The final suture line is not parallel to line of lengthening. These are probably the reasons for no recurrence. In the developing world scenario like ours, patients come from poor socioeconomic groups, often travelling from distant regions and are not always compliant with prolonged splinting protocols and follow ups. In square flap technique, postoperative splints were used only for one week. The axillary hair patch was not cut across and displaced giving a better cosmetic appearance. The only complication seen in our series was tip necrosis of a flap, probably due to poor vascularity of the adjacent scar tissue on which the triangular flap was raised. 

Square flap was shown to be a simple technique and easy to replicate. It gives good lengthening and requires less post-operative splinting with no donor site morbidity and a good cosmetic outcome. In our experience, square flap is a reliable local flap technique for mild to moderate axillary contractures of the anterior or posterior axillary folds even when there is significant adjacent scarring of chest wall or back (types I and II). 
